# Identity under threat: internal conflict and stigma in undetected child sexual abuse material users

**DOI:** 10.3389/fpsyt.2026.1830667

**Published:** 2026-07-31

**Authors:** Lucia Nemcová, Adam Schreier, Tereza Karousová, Lucie Kalenská, Gabriel Bianchi, Maria Breide, Stina Lindegren, Rafael Ballester-Arnal, Peer Briken, Michal Chovanec, Sophie Dixelius, Marcel Elipe-Miravet, Lisann Högström, Johan Holmberg, Tarik Korkutan, Jozef Metenko, Sabine Prantner, Ellen Zakreski, Christoffer Rahm, Kateřina Klapilová

**Affiliations:** 1 Faculty of Humanities, Charles University, Prague, Czechia; 2 Centre for Sexual Health and Interventions, National Institute of Mental Health, Prague, Czechia; 3 Faculty of Arts, Charles University, Prague, Czechia; 4 Institute for Research in Social Communication, Slovak Academy of Sciences, Bratislava, Slovakia; 5 Centre for Psychiatry Research, Department of Clinical Neuroscience, Karolinska Institutet & Stockholm Health Care Services, Stockholm, Sweden; 6 Department of Social Work, Uppsala University, Uppsala, Sweden; 7 National Center for Knowledge on Men’s Violence Against Women, Uppsala University, Uppsala, Sweden; 8 Universitat Jaume I, Castellon, Spain; 9 University Medical Center Hamburg-Eppendorf, Center for Psychosocial Medicine, Institute for Sex Research, Sexual Medicine & Forensic Psychiatry, Hamburg, Germany; 10 Academy of the Police Force in Bratislava, Bratislava, Slovakia

**Keywords:** compulsive sexual behavior, CSAM users, cultural formulation interview, ego threat, patient centered approach, pedophilia, stigma, thematic analysis

## Abstract

**Introduction:**

Individuals who use child sexual abuse material (CSAM) represent an under-researched population, particularly those who remain undetected by the legal system and outside formal treatment settings. Existing research is predominantly expert-driven and rarely captures users’ subjective experiences or sociocultural contexts. This lack of perspective limits the development of patient-centered and culturally responsive prevention strategies.

**Methods:**

This study utilized a qualitative design to analyze anonymous, chat-based Cultural Formulation Interviews (CFI) conducted within the EU-funded Project Bridge. The sample consisted of 32 help-seeking CSAM users assessed as low-to-medium risk for hands-on offending, representing four language groups (English, Swedish, Czech, and Finnish). Data were analyzed using reflexive thematic analysis to capture the "user-only" perspective.

**Results:**

CSAM use emerged as a deeply distressing and heterogeneous experience shaped by a perceived loss of control, internal conflict, identity threat, and experienced stigma. Participants’ explanations of causality (e.g., innate, learned, or addiction-like accounts) functioned as meaning-making strategies to negotiate shame, responsibility, and self-concept, which occasionally included denial of responsibility or socially desirable responding. While secrecy, social isolation, and limited access to support acted as major barriers to help-seeking; relational stability, emotional well-being, and structured life circumstances functioned as key facilitators that supported and regulated engagement with support services.

**Discussion:**

These findings demonstrate the critical value of informed, patient-centered qualitative approaches for understanding CSAM use beyond traditional diagnostic and risk-focused models. Methodologically, adapting the CFI to an anonymous, chat-based format represents a vital innovation for accessing hard-to-reach populations fearing social condemnation. Culturally sensitive, low-threshold interventions must be designed to address ambivalence, stigma, denial, and contextual life factors as core components of effective prevention and support.

## 1 Introduction

Child sexual abuse material (CSAM) refers to visual content depicting children in sexualized contexts or engaged in sexually explicit conduct, primarily for sexual purposes (1). CSAM constitutes one of the most prevalent forms of child exploitation online (2), reflected in the scale of global reporting. For example, the National Center for Missing & Exploited Children (3) reported receiving more than 35 million tips concerning suspected CSAM within a single year. Although such material is accessible through the conventional internet (“clear web”), a substantial proportion is also distributed via the “dark web,” a concealed part of the internet requiring specialized software for access (4).

CSAM use constitutes a serious criminal offense that can cause profound harm to children and may increase the risk of contact sexual offending (5). The production and distribution of such material inflict severe and enduring psychological trauma on victims, often manifesting as post-traumatic stress disorder, chronic anxiety, and disruptions in identity development (6). Furthermore, because these materials can be disseminated indefinitely across digital platforms, survivors are subjected to what has been described as “continuous victimization,” where the ongoing circulation of their abuse and the loss of control over its visibility significantly hinder recovery (7). While the negative impact on victims remains a constant reality of this material, the psychological drivers that lead individuals to consume CSAM are heterogeneous, necessitating a move beyond simplistic labels. Public discourse often equates CSAM use with pedophilia (8), although existing research indicates that CSAM use may be associated with a diverse array of motivational and facilitative factors beyondpedophilic interest, such as compulsive sexual behavior, curiosity, arousal by the taboo and/or forbidden, coping with stress, lack of insight into the impact and consequences of CSAM use, and communal reinforcement within online spaces (9–11). It is thus important to recognize CSAM users as consisting of different subgroups.

At the same time, consumers of CSAM across subgroups often struggle to disclose their CSAM use or seek help, even when experiencing distress and a desire to stop (9, 10, 12). This is particularly important given the current shift toward proactive intervention and the need to build strategies for primary and secondary prevention of CSAM use (13, 14). Preventive clinical interventions should be understood as complementary to law enforcement and technological measures, which remain the primary societal responses to detecting and disrupting CSAM-related offenses (4, 13).

Although research into the typology and healthcare needs of CSAM users is expanding, many studies overlook the individual’s subjective experience and the broader sociocultural contexts that influence help-seeking behaviors. Furthermore, existing research often targets individuals processed through the legal system (8, 10), leaving undetected populations understudied. Current low-threshold interventions are also typically used internationally without further cultural adaptation other than translation into different languages (Breide et al., 2026 [in prep]; Insoll et al., 2026 [in prep]; 15), implicitly assuming minimal cultural variation among target users from different sociocultural contexts.

The present study seeks to address this gap by using the Cultural Formulation Interview (CFI) to investigate CSAM users across four European language groups and to examine how the European sociocultural context influences their internal conflicts and their subjective problem perception. This general approach aims to inform broader international treatment strategies and online interventions, specifically those developed within the Bridge project (Breide et al., 2026; Insoll et al., 2026; 16). In this paper, we strive to do so by interviewing low-to-medium risk individuals in continental Europe, who have a sexual interest in minors, use CSAM, and have entered the Bridge project, to explore how these individuals 1) specifically perceive and describe their CSAM-related problems, and 2) explore what causes and contexts of the problem they perceive.

## 2 Literature review

### 2.1 CSAM use, heterogeneity, and motivations

Recent empirical findings suggest that the motivations behind CSAM use are significantly more heterogeneous than popular discourse acknowledges. Public discourse commonly equates CSAM consumption with pedophilia (8). Some users indeed describe their problem in accordance with the definition of pedophilic disorder, as persistent sexual interest in prepubescent children (17). However, sexual interest in children and sexual offending are two distinct constructs which might not always overlap (18). Previous evidence shows only some CSAM users have pedophilic interest varying from 49.4% (19), 60% (20) to 85% (21) depending on the research methods and population targeted; others present with patterns of compulsive sexual behavior that culminate in CSAM consumption (9–11).

Besides sexual interest in children, other known motivators for CSAM use are initial curiosity, arousal by the taboo and/or forbidden, seeking an escape from reality and/or coping with real-life stress through CSAM consumption (9, 10), lack of insight into the impact and consequences of CSAM use, and communal reinforcement within online spaces (9).

### 2.2 Help-seeking barriers in CSAM users

While motivations behind CSAM use vary, the struggle to share their problem or seek help stays relevant across all subgroups even when distressed and when they express a desire to stop (9, 10, 12). A common denominator among CSAM users with pedophilic interests is the fear of seeking help directly associated with their sexual attraction to children (22, 23). Across all subgroups of CSAM users, research suggests a constant struggle where the reasons to seek help are often directly tied to the very things that make people afraid to reach out. These include fear of exposure by professionals and other consequences associated with their problem being made public (e.g., job loss, social exclusion, loss of family and friends, imprisonment, being attacked), fear of being reported; being misunderstood by the therapist; overwhelming shame and guilt associated with their problem; but also antisocial behaviour, social desirability, and the fear of punishment (9, 10, 12, 22–26).

### 2.4 Prevention-oriented responses to CSAM use

Identifying and understanding the non-forensic population is critical given the current shift toward proactive intervention. The vast majority of recent expert and scientific efforts emphasize the need to build strategies for primary and secondary prevention of CSAM use. Primary prevention targets potential offenders before any offense occurs (14, 27), while secondary prevention aims at early detection and education to reduce harm and offer help (27). Preventive clinical interventions should be understood as complementary to law enforcement and technological measures, which remain the primary societal responses to detecting and disrupting CSAM-related offenses (4, 27). Using a public health lens (14), these interventions complement rather than replace existing strategies by reaching distressed, conflicted individuals who might otherwise go unnoticed.

For these strategies to achieve their intended reach, it is imperative to integrate the target population’s self-understanding of their problems and their specific motivations for and fears of accessing support.

### 2.5 Current interventions and the need for person-centered insight

To date, several online help programs for CSAM users have emerged within the secondary prevention framework (for review, see 28). Low-threshold self-help, for example ReDirection, Stop It Now! self-help modules (29), Troubled Desire (15), and Bridge (Insoll et al., 2026 [in prep]), and therapist-assisted online programs, for example Troubled Desire (15), Inform Plus or Engage Plus (29), Prevent It (30), and Bridge (Breide et al., 2026 [in prep]), show promise, with some even demonstrating effectiveness via RCTs, such as PRIORITY (30). Creative approaches have also been implemented to reach and motivate this target group to engage in care, including public health campaigns (31), pop-up deterrence messages deployed on clearnet erotic servers (32), and similar interventions on the dark web (4).

These interventions however, remain largely expert-driven and do not sufficiently incorporate the perspectives and lived experiences of CSAM users themselves. To move beyond a one-size-fits-all approach to CSAM interventions, it is necessary to to gain qualitative insight into the unique motivational states, internal conflicts, and individual needs that influence decisions to seek help in such a hard-to-reach and understudied population. Such insight could facilitate the adaptation of suggested treatment approaches to make them more individualized, reduce dropout, and increase compliance.

### 2.6 Sociocultural context and cultural adaptation of interventions

Individualizing interventions is further complicated by the fact that CSAM use does not occur in a vacuum; rather, it is deeply embedded in diverse sociocultural and legal landscapes. The hidden nature of CSAM and the difficulty in reaching CSAM users predetermine many challenges stemming from sociocultural differences even within the European context, ranging from different crime prevention systems as well as from varying levels of access to health care across countries (33, 34). Countries also differ in their definitions of sexual offences and the extent of mandatory reporting obligations, which further complicates the cultural adaptation of preventive interventions.

While further research on the cultural dimensions of child sexual abuse within Europe remains limited, evidence from other regions clarifies how specific value systems can exacerbate these structural barriers. In Asian American family systems, values such as collectivism, hierarchy, filial piety, family honor, and the desire to avoid “loss of face” may create systemic barriers to disclosure and recognition of child sexual abuse (35). Similarly, Shafe and Hutchinson (36) note that collectivist orientations sustain silence and that practices such as child marriage and beliefs treating children as parental property further heighten vulnerability to abuse.

From a broader global perspective, Buller et al. (37) conducted a large-scale systematic review spanning 37 countries and identified six cultural norms that perpetuate sexual exploitation: materialism and the pursuit of status, social pressure to be sexually active, community expectations of economic contribution, stigmatization of victims, social tolerance of perpetrators, and the absence of effective sanctioning mechanisms. Shafe and Hutchinson (36) reinforce this perspective, demonstrating that cultural constructs shape both the occurrence and concealment of child sexual abuse globally. This includes the aforementioned cultural practices, as well as, for example, religious and patriarchal hierarchies that legitimize power imbalances and weaken reporting systems. Although this literature does not directly address CSAM users’ help-seeking, it demonstrates that culturally shaped meanings may influence how child sexual abuse is understood, disclosed, concealed, and responded to.

Despite these documented cultural nuances, currently available low-threshold interventions for CSAM users are typically used internationally without cultural adaptation other than translation to different languages (Breide et al., 2026 [in prep]; Insoll et al., 2026 [in prep]; 15). This implicitly assumes minimal cultural variation among target users from different sociocultural contexts. To our knowledge, very few culturally sensitive tools or qualitative methodologies have been applied to capture user-relevant differences that shape problem perception, needs, and barriers to care among CSAM users.

The existing models of cultural adaptation of online interventions in general emphasise that such programs may require both surface-level adjustments, such as language, and deeper structural changes that reflect cultural concepts of distress, values, norms, treatment expectations, and contextual determinants of behaviour (38–40). It was repeatedly found that cultural adaptation of psychological interventions may increase compliance to interventions and its effectiveness (41–43), with a few attempts for cultural adaptation in the field of sexual health (44, 45).

These challenges indicate the need for a method capable of capturing not only individual distress and motivation, but also culturally shaped meanings, expectations of care, and perceived risks of disclosure.

### 2.7 The cultural formulation interview as a methodological tool

One valuable tool for systematically capturing relevant sociocultural differences in diagnostic methods and interventions is the Cultural Formulation Interview (CFI; 46). The CFI is a validated standardized, semi-structured interview recommended by the American Psychiatric Association (47) that facilitates the exploration of how individuals understand, experience, and contextualize their problems within their unique cultural and social environments. It is widely used in both clinical practice and research to promote culturally sensitive, person-centered care (48, 49).

The CFI has demonstrated strong feasibility and usability in face-to-face healthcare settings. For instance, it has been successfully applied in the assessment of eating disorders (50, 51), with non-native speakers in Swedish psychiatric services (52, 53), and in transgender and gender-diverse healthcare (54). However, it has not been adapted for or evaluated in low-threshold or fully online interventions.

In the present study, the CFI is therefore used as a person-centered framework for eliciting culturally situated accounts of CSAM-related problems.

## 3 Theoretical framework

Several theoretical frameworks can help illuminate how sociocultural factors shape the ways in which CSAM users perceive, interpret and narrate their CSAM-related problem, including the perceived cause, contexts, and consequences of their behavior. CSAM users’ perspectives can influence how the behavior is understood, how risk and responsibility are defined, and what types of support or intervention are considered appropriate. The following section introduces the main interpretative frameworks used in this paper.

Together, these frameworks guide the interpretation of three analytically distinct dimensions of CSAM users’ narratives: 1) stigma and fear of seeking help, 2) motivational and situational pathways to CSAM use, 3) and the distinction between clinical, subjective, and socially constructed meanings of the problem.

### 3.1 Stigma and stigma-related stress

Stigma and internalized stigma (autostigmatization) represent one of the most powerful sociocultural barriers reported by this population. According to Goffman (55), stigma refers to a socially constructed attribute or characteristic that discredits a person in the eyes of others. This creates a significant gap between how individuals see themselves and the devalued identity that society assigns to them. When people absorb these negative societal views, it results in autostigmatization (56), which can seriously damage their self-concept and psychological well-being. Among groups with paraphilic interests, those with sexual interest in children are the most heavily stigmatized (57). Building on the concept of internalized stigma, Elchuk et al. (58) introduced the term internalized pedonegativity to describe the internalization of stigmatizing beliefs about pedophilia by individuals with pedophilic interests; according to the author, higher levels of internalized pedonegativity have been associated with lower perceived social support, greater feelings of loneliness, increased psychological distress, and heightened suicidality.

The stigma-related stress model (59) explains stigma as a chronic social stressor harming emotional well-being, introducing the themes of shame, guilt, and fear of reaching out (60, 61). The model distinguishes external stressors, such as discrimination and rejection, from internal processes, such as anticipated rejection, identity concealment, and internalized negativity. Applied to pedophilic individuals, societal condemnation and fear of exposure create ongoing stress that contributes to depression, anxiety, isolation, and reduced willingness to seek help. Chronic stress not only deteriorates mental health, but also increases the risk of sexual offending (62).

### 3.2 Motivation-facilitation model of sexual offending

In addition to stigma-related frameworks, several CSAM-specific explanatory models of sexual offending have addressed why individuals use CSAM. An early model of problematic Internet use moved beyond explanations of CSAM use as either “addiction” or a direct extension of contact offending, emphasizing instead how cognitions can support access to illegal material, sustain Internet engagement, and normalize continued use (63). A separate classification model distinguishes CSAM users according to three dimensions: whether the offending is fantasy-driven or contact-driven, what motivates the use of CSAM, and the degree of situational and social engagement in the offending behaviour, including networking with other users (64). A later case formulation model shifts the focus from classification to individualized assessment, linking CSEM offending to developmental context, psychological vulnerabilities, personal circumstances, permission-giving thoughts, Internet behaviour, perceived consequences, and desistance processes (65). Other work suggests that onset and maintenance may follow different motivational pathways, particularly sexual gratification and avoidance of emotional pain, while also allowing for escalation or behavioural facilitation through repeated exposure to Internet pornography (66). More recent technology-focused work shows that online environments are not merely passive access points: individuals may select spaces that meet psychosexual needs with low friction, while habituation, perceived anonymity, social reinforcement, and reduced perceived guardianship can make continued offending easier to maintain (67).

For this study, we adopted the widely used Motivation–Facilitation Model of Sexual Offending, which conceptualizes sexual offending, including CSAM use, as the outcome of interactions between motivational, facilitative, and situational factors which can vary by individual (62). This model was selected because it has been explicitly extended to child pornography offending and online solicitation, and because it is well suited to the heterogeneity of CSAM users. In Seto’s model, primary motivational factors for sexual offending include paraphilic interests, high sex drive, and intense mating effort, while facilitative factors include trait factors such as antisociality, self-regulation problems, and hostile masculinity, as well as state factors such as intoxication and negative affect (e.g., anger, stress). Situational factors, including access to children or to CSAM, Internet technologies, perceived anonymity, the absence of capable guardianship, and broader contextual opportunities, further shape whether and how motivated individuals engage in offending behaviour.

This framework is particularly relevant for interpreting qualitative accounts of CSAM users that emphasize internal conflict, fluctuating control, and context-dependent behavior rather than stable offending trajectories. In relation to CSAM offending, Seto argues that individuals who use child sexual abuse material may have pedophilic or hebephilic sexual interests, but often show lower antisociality, higher self-control or fewer facilitating factors, greater access to Internet technologies, and less access to children than individuals who committ contact sexual offenses against children (62, 68).

### 3.3 Offence-supportive cognitions

To further understand how these individuals interpret and explain their behavior, the present study also draws on theories of offence-supportive cognitions. Offence-supportive cognitions are beliefs or ways of thinking that excuse, justify or minimize sexual offending (69, 70). These cognitions can increase the risk of sexual offending, and are consistent with the definition of a facilitative factor in the Motivation-Facilitation model (62). While early models conceptualized offence-supportive cognitions as relatively stable belief systems (70, 71), more recent approaches emphasize their dynamic, situational, and regulatory nature (72, 73). Specifically, offence-supportive cognitions may form after sexual offending to reduce shame and preserve self-image, form before offending to disinhibit the individual and allow them to obtain something they want, or they can form as a result of childhood trauma, as part of a stable belief system, for example the world is hostile or adults are untrustworthy (73). From this perspective, offence-supportive cognitions may function as meaning-making strategies that help manage moral conflict, stigma, and perceived loss of control. This conceptualization is particularly relevant for qualitative research, as it allows participants’ explanatory narratives to be interpreted as adaptive responses to distress and social condemnation (73, 74).

### 3.4 Disease-illness-sickness framework

A final framework helps distinguish between different social and clinical layers of CSAM-related experience. The disease-illness-sickness framework, originally proposed by Nordenfelt and Twaddle (75), separates three analytically distinct frameworks: 1) disease (the clinically defined biomedical condition), 2) illness (the subjective lived experience), and 3) sickness (socially constructed meanings and moral evaluations) Costa and Sera (76) have demonstrated the value of this triadic model as an integrative interpretative framework, particularly for complex and contested phenomena where biomedical, experiential, and sociocultural dimensions are routinely conflated. Rather than serving as a diagnostic model, the triad is used to clarify levels of analysis and to prevent errors in interpretation.

The disease-illness-sickness is relevant for the study of pedophilia and CSAM-related experiences, where social condemnation (sickness), diagnostic classification (disease) and lived distress and self-regulation (illness) are often collapsed into a single undifferentiated explanatory narrative. Because CFI elicits participants’ own explanatory narratives, the disease-illness-sickness framework helps distinguish whether a given narrative reflects clinical symptoms, subjective distress, or socially imposed meanings. As shown by Costa and Serra (76), applying the disease-illness-sickness allows researchers to disentangle moral and social judgments from subjective suffering and clinical reasoning, thereby improving conceptual clarity and interpretative rigor. In the present study, the disease-illness-sickness framework is therefore used as an epistemological lens to guide the interpretation of qualitative accounts, ensuring that participants’ narratives are analyzed at the appropriate level and that stigma-related meanings are not uncritically translated into psychopathological claims.

## 4 Methods

Project Bridge comprised three parallel studies conducted in two phases: a pilot phase (30.6. - 3.9.2023) and a randomised controlled trial phase (18.9.2023-21.6.2024; Karolinska Institutet, NCT06(133569)). Two studies evaluated low-threshold interventions for individuals concerned about sexual urges involving children who use CSAM: MI Bridge, a motivational interviewing intervention designed to increase help-seeking among high-risk individuals, and ReDirection, a self-help program supporting low–medium risk individuals in stopping CSAM use (Insoll et al., 2026 [in prep]; Breide et al., 2026 [in prep]). A third qualitative study used the Cultural Formulation Interview to examine how cultural factors shape problem understanding and help-seeking, with the aim of informing culturally adapted interventions. This paper draws on those interview data collected at intervention entry. This means that at the time of the CFI interview, participants had not yet received any intervention and had yet not received any tested intervention. Data were collected across six European sites: the Czech Republic, the Slovak Republic, Sweden, Finland, Germany, and Spain.

All studies were approved by the Swedish Ethical Review Authority (Dossier No. 2023-02321-01) and by local ethics committees. Participants agreed to informed consent which included data management and publishing. Data was stored on secure servers at Karolinska Institutet, Stockholm. Participation in the Cultural Formulation Interview was anonymous; participants were instructed not to share identifying details, and interviews were further pseudoanonymised prior to analysis.

Participants were recruited internationally through clear web and dark web channels. Recruitment included a Google Ads campaign linking to the project website and a dark web campaign via an encrypted onion link. Advertisements were placed on the Ahmia.fi dark web search engine, which restricts results to research and support resources when child sexual abuse-related keywords are used (4). Additional national recruitment campaigns were done depending on site capacity [16; Insoll et al., 2026 (in prep)].

The inclusion criteria for participation in the project were age of at least 18 years; concern about one’s own sexual urges involving children; CSAM use in the past six months; and sufficient language skills in English, Spanish, German, Finnish, Swedish, Czech, or Slovak. Exclusion criteria were presence of severe psychiatric illness and refused to participate. Excluded participants were referred to the healthcare or other available interventions.

### 4.1 Design

Participants were informed not to share any identifying information and that the project was fully anonymous. Written informed consent was obtained prior to registration for a chat-based screening interview. At the end of the screening, participants were invited to participate in the CFI and informed that declining would not affect their involvement in the project.

Chat based interviews were conducted using the semi-structured CFI tool (77). Following the CFI, participants completed the baseline measurement, including the questionnaire SChiMRA+ (78) assessing the participants’ risk level to commit child sexual abuse. This means the interviewers were unaware of the participants’ risk levels during the interview and that at the time of the CFI interview, participants had not yet received any Bridge intervention. Prior engagement with mental health or other professional services before study entry was not an exclusion criterion and was explored within the CFI protocol; however, findings related to prior help-seeking are not reported in the present study.

A formal safety protocol was in place throughout data collection. Participants were informed of mandatory reporting obligations prior to the screening interview. The interviewers were required to follow the mandatory reporting legislation of their country of practice if a participant disclosed intent to commit a criminal offense or revealed a past offense subject to mandatory reporting. Regarding participant safety, a structured suicide risk protocol was followed if severe suicidal ideation emerged during the interview. This included expressing concern, encouraging the participant to seek appropriate local care, providing relevant crisis service contacts where possible, and consulting with the local principal investigator for further risk assessment. For acute suicidal risk, referral to emergency psychiatric services was indicated.

### 4.2 The cultural formulation interview protocol

The CFI interview protocol is designed to explore cultural and linguistic specificities in participants’ perceptions of their problem and help-seeking behavior^1^. The interview consists of 16 questions divided into four key domains: 1) Cultural definition of the problem, 2) Cultural perception of cause, context, and support, 3) Cultural factors influencing self-coping and past help-seeking, and 4) Cultural factors influencing current help-seeking. The interview enables an understanding of the cultural meanings of health, illness, and treatment from the participants’ perspectives (48).

The CFI interview protocol (77) refers to culture as follows: “*Culture refers to systems of knowledge, concepts, rules, and practices that are learned and transmitted across generations. Culture includes language, religion and spirituality, family structures, life-cycle stages, ceremonial rituals, and customs, as well as moral and legal systems. Cultures are open, dynamic systems that undergo continuous change over time; in the contemporary world, most individuals and groups are exposed to multiple cultures, which they use to fashion their own identities and make sense of experience”* (77). We follow this definition of culture throughout the paper.

To illustrate the cultural focus of the interview, Domain 1 (Cultural definition of the problem) included questions such as “Sometimes people have different ways of describing their problem to their family, friends, or others in their community. How would you describe your [PROBLEM]?”, encouraging participants to describe their problem in their own terms and drawing on culturally specific language and meaning. Domain 2 (Cultural perception of cause, context, and support) included questions such as “Are there any aspects of your background or identity that make a difference to your [PROBLEM]?”, inviting reflection on how cultural background, community norms, and social environment shaped participants’ understanding of their situation. The problem in questions was substituted with what participants identified as the problem bringing them into the project, for example CSAM consumption.

In this study, the CFI was translated into six languages following recommendations from the author of the tool: using forward translation by a bilingual speaker, followed by review and item-level discussion by a bilingual consensus committee, including *ad hoc* back-translation where needed (Lewis-Fernández, personal communication, March 2023).

The feasibility of the adapted CFI interview protocol was assessed during the pilot phase of the Bridge project, from June 30, 2023 to September 1, 2023, and consisted of 11 CFI interviews in total. The final version included the 16 original core questions and the corresponding prompts from the CFI, supplemented by a modified component consisting of 13 selected questions from the CFI supplementary modules and eight alternative wordings of the core questions (see *The CFI adapted* in **Supplementary Materials**). All interviewers underwent a specialised online training module (48) provided by the New York Center of Excellence for Cultural Competence (https://nyculturalcompetence.org/). Training was followed by practice via online sessions to ensure consistency in applying the CFI protocol across all sites.

For the purpose of this paper, only the first two domains of the protocol were used concerning the cultural definition of the problem in Domain 1 and Cultural perception of the cause, context and support in Domain 2. If the core questions did not elicit elaborate answers, the interviewer could prompt further using supplementary questions or alternative phrasing (77).

Interviews were conducted by trained and supervised interviewers from each involved institution in their native language; Czech, Slovak, German, Finnish, Swedish, Spanish or in English. During analysis, the research team did not have the necessary language resources to conduct in-depth analysis of the Spanish and German interviews, and these were therefore not included in this paper.

In the current study, the CFI was used as a chat interview for the first time in research. For these purposes, a manual for conducting the CFI in the project Bridge setting was created and translated into the local languages of the involved study sites (available upon request). The interviews were conducted and stored in chat format within the online treatment platform Iterapi, and later on downloaded and stored in the protected institutional repository after the end of the RCT period.

### 4.3 Risk-assessment measures

Participants’ level of risk of sexual offending and relevant clinical symptoms were assessed using a self-report questionnaire battery that was assigned to participants after completing the CFI interview. This precaution was taken to minimise the influence of study procedures on participants’ responses during the CFI.

Risk level was determined using the online version of the *Sexual Child Molestation Risk Assessment* (SChiMRA+). Part A assesses self-reported likelihood of engaging in sexual behaviour with children if they would not be detected, while Part B captures behaviours motivated by sexual interest in children during the past seven days (78, 79). Participants were classified as low–medium risk if they scored 3 or lower out of 10 on Part A item 2 or 3 and declined any socializing or sexual interaction with children on Part B items 2 and 3 (Insoll et al., 2026 [in prep]), participants with higher scores were classified as high risk (Breide et al., 2026 [in prep]). In this study, only CFI interviews of low-medium risk CSAM users were analyzed because of the space constraints.

### 4.4 Sample

From August 18 2023 to July 21 2024, 123 CFI’s were conducted. Because recruitment was done via the dark web, participants could join the study from various continents; however, their specific location or country of residence could not be tracked. Despite the CFI being voluntary, in the project workflow, 123 participants out of the 229 who completed the screening interview agreed to participate, representing 46.3% drop out.

Out of the total 123 respondents who underwent the CFI interview, 81 reported Europe as their place of residence. Specifically, 9 interviews were conducted in Czech, 22 in German, 2 in Finnish, 12 in Swedish, 3 in Spanish and 33 in English. No Slovak speaking participant registered for the CFI. Additionally 42 respondents stated another continent as their place of residence. See the distribution of the CFI participants according to their risk level in **Table 1**.

**Table 1:** Overview of the number of CFI interviews conducted by continent and language (interviews utilized in present analysis are indicated **bold**).

The number of CFI		N RCT by risk level		
Continent	Language	n HR	n L/MR	Not idenfied
Europe	Czech	2	**7**	0
	German	4	18	0
	Finnish	1	**1**	0
	Swedish	6	**6**	0
	Slovak	0	0	0
	Spanish	1	2	0
	English	12	**18**	3
Sub-total		26	52	3
	European	81		

For the purposes of the current thematic analysis, only interviews from participants who reported residing in Europe (n = 81) were considered due to higher cultural coherence (80). For consistency in analytic procedures, only interviews conducted in languages for which a full thematic analysis capacity was available were included. Specifically, we have included interviews conducted in Czech, Finnish, Swedish, and English. German and Spanish language interviews were excluded at this stage. Interviews from respondents without an identified risk level were also excluded. Overall, 53 interviews were analyzed using thematic analysis, 21 from high risk participants and 32 from low-medium risk participants. This article focuses exclusively on data from participants assessed as low-medium risk (n = 32; indicated in bold in **Table 1**). Due to the extent of the data analysis, findings from high-risk participants will be presented in forthcoming articles. The average length of the interview in the low-medium risk group was 2 hours 7 minutes representing on average 7 pages of the text.

### 4.5 Thematic analysis

Thematic analysis by Braun & Clarke (81) was adopted as the analytical approach and a manual was created to aid coders through the coding process (available upon request). TA is a method for identifying, analyzing, and reporting themes within data (81). It has been chosen because it represents a flexible way to identify, analyze, and document patterns (or themes) across the available materials, while it also provides a clear structured approach that helps to minimize the risks of preconceived notions on the researcher’s part contaminating the results (81, 82).

Researchers in each of the participating countries carried out thematic analysis based on the analysis manual developed based on Braune and Clarke (81) as well as descriptive examples of each phase (83). The analysis was carried out using the qualitative software ATLAS.ti version 25.0.1. (84). The semi-structured interviews were qualitatively analyzed collaboratively across four participating language groups (English, Swedish, Czech and Finnish) to avoid methodological limitations associated with translating interviews into English (see also 85). Eight researchers participated in the analysis of Czech, Finnish, Swedish, and English interviews. The researchers analysed the interviews in pairs, taking into account their native language and ensuring reliability through ongoing verification of the findings but prepared the outcome materials in English to enable a full group discussion over findings.

The analytic process followed the six phases of reflexive thematic analysis as outlined by Braun and Clarke (81). The dataset was divided by language group, with each group assigned a dedicated coding dyad; English-language interviews were distributed across dyads to ensure an approximately equal workload.

In the first phase (familiarization), all coders repeatedly read their assigned interviews before beginning any coding to develop thorough familiarity with the data. In the second phase (generating initial codes), each dyad independently coded two shared interviews before meeting to compare, discuss, and merge their codebooks into a shared framework. No formal inter-rater reliability statistics were calculated; the collaborative coding discussion was intended to deepen analytical reflection and align interpretive perspectives rather than to establish quantifiable agreement between coders. Coders subsequently worked independently using the shared codebook, maintaining close contact throughout to discuss emerging codes and questions.

In the third phase (constructing themes), each dyad generated preliminary themes based on the acquired codes. In the fourth phase (reviewing and developing themes), these preliminary themes were presented and discussed across all dyads in weekly supervision sessions, enabling cross-group alignment and ongoing refinement. An additional consultation with an external qualitative methods expert was conducted to further develop the emerging thematic structure. Each dyad compiled an initial report including theme definitions with illustrative code examples (available in **Supplementary Material**). Throughout the analytic process, ongoing consultation with the Czech principal investigator (K.K.) provided continuous critical oversight of the developing thematic structure.

In the fifth phase (refining, defining and naming themes), the Czech research team, which held analytical leadership of thematic analysis within Project Bridge, conducted a cross-dyad synthesis identifying shared themes across all language groups. The resulting thematic structure was submitted to peer validation by the original coding dyads, and subsequently refined and finalised in response to their feedback. In the sixth phase (writing up), the final integrated themes, collaboratively validated across all dyads, are presented in the current article.

## 5 Results

To provide a comprehensive overview of the outcomes of our thematic analysis we have integrated themes across all the coding dyads (see **Table 2**). Together, they form 8 overarching themes (“CSAM use as a conflict”, “Lived experience of fear, anxiety, and distress”, “Ego threat”, “Sensation Seeking and Compulsion”, “Innate versus Learned”, “Unspeakable Topic: The Internalised Taboo”, “Psycho-Social Regulators of Use: A Unique Life-Coping Strategy” and “Stigma and Social Exclusion: Shame, Silence, and the Loss of Connection”; see **Supplementary Figure 1**.) described below in detail with illustrating quotes from the chats. To illustrate the themes, quotations of participants’ statements are provided in italics.

**Table 2:** Themes and corresponding domains.

Content of the domain	Themes	Subthemes
Perception of the problem	CSAM as a Conflict	Intrapsychic conflict
		Social conflict
	Lack of Control: Living with Fear, Anxiety and Distress.	Negative emotions and distress
		Lack of control
		Addiction-like narrative
	Ego Threat	Self-esteem reduction
		Normality
		Rationalization of CSAM consumption to protect self-image
		Responsibility reduction to protect self-image
	Sensation Seeking: A Compulsive Justification	
Perceived cause and context	Innate versus Learned	Innate
		Learned
		Inability to find cause
	Unspeakable Topic: The Internalised Taboo	
	Psycho-Social Regulators of Use: A Unique Life-Coping Strategy	
	Stigma and Social Exclusion: Shame, Silence, and the Loss of Connection	

For detailed visualisation of the common themes and how these manifested in each language group see **Supplementary Figure 2**. *Description of the problem, across coding dyads* and **Supplementary Figure 2**. *Perceived Cause and Context, across coding dyads* in **Supplementary Material**. For the comprehensive reports with definitions of themes, used codes and interview extracts from each language group see **Supplementary Material 2***. Low-medium risk, Report Thematic analysis*.

### 5.1 Description of the problem

#### 5.1.1 CSAM use as a conflict

This theme captures participants’ intrapsychic and social conflict: the tension between their sexual urges and their desired self-image and/or societal values. It was common for participants to feel stuck between these opposing forces, as they could not satisfy both at the same time, which caused further distress. The societal part of the conflict is related to the perceived stigmatisation, which often manifests as feelings of shame. The sexual urges and self-image disruptions manifest as feelings of guilt and have an impact on their self-esteem, possibly leading to self-stigmatisation. The feelings of guilt and self-stigmatization were often framed within culturally embedded moral and social norms.

##### 5.1.1.1 Intrapsychic conflict

Participants often reported an ongoing internal struggle as they attempted to resist their sexual impulses and urges toward CSAM. They recognized that acting on these urges would be morally wrong. Some respondents also perceived themselves as “evil” and had a notion that they were doing wrong things. Many acknowledged that their CSAM use involved hurting children and that this is something they do not want to do as it conflicts with their morals. While they were usually able to acknowledge the harm they were causing or participating in, the degree of their awareness varied.

“[mostly bothers me] … several different things. That I view children being harmed, that the material was created on the premise that a child is in the hands of an adult man, and sexual and often also financial interests for this person, and that the child is just an object.”]

Such reflections were sometimes accompanied by intense emotional distress and periods of acute psychological crisis:

“Basically I had a mental health crisis and started panicking over how many of my behaviors over the years could have harmed people, and needed to figure out a way to live with myself because I know that I can’t just undo things.”

At the same time, participants described experiences in which urges to use CSAM felt overpowering and difficult to control.

“I get an urge, which completely takes over me. I am unable to stop myself. Which leads me to do things I don’t want.”

Participants in general described an ongoing tension between moral awareness of harm and recurrent experiences of overpowering urges further discussed in the theme Lack of control.

##### 5.1.1.2 Social conflict

Participants described struggling with being perceived as evil in the eyes of others and anticipated strong moral judgments for behavior that conflicts with social norms to a severe degree, as it directly harms children and contributes to their victimization. Many expressed fear of shame and condemnation:

“[I feel] shame, judgment - I don’t want to know what people would think of me, what they’d say to me.”

These types of anticipated judgement were closely linked to fear of relational loss:

“ I’m afraid of losing them [my loved ones]”

While others expected broader social consequences if their behaviour would be discovered.

“I’d expect to be excluded from society.”

Alongside fears of rejection and exclusion, some participants described ways in which they unknowingly downplayed the severity of their behavior in relation to prevailing social norms. In rare cases the severity of this behaviour was striking:

“I occasionally watch some pornographic content with the people below 16 in Darknet, but I don’t consider it as a problem. (…) I presumably concerned with the fact that society consider it harmful and amoral.”

In other accounts, participants acknowledged the broader societal view and the harm of CSAM perpetration but erroneously framed their own involvement as limited to individual actions:

“…I’m not doing any harm by that since I technically do not influence anything on my own in this regard (I never pay for it or distribute it myself even), but society thinks otherwise.”

Social conflict was thus described both as fear of rejection, exclusion and moral condemnation, and as a tension between personal justifications and anticipated societal judgement.

#### 5.1.2 Lived experience of fear, anxiety, and distress

##### 5.1.2.1 Negative emotions and distress

This theme captures CSAM use experienced as losing control over one’s thoughts, emotions and behavior. A core aspect of this experience was the chronic persistence of fear, anxiety, and distress that sometimes took control over participants’ daily lives. Participants lived in a constant and pervasive state of fear related to the possible discovery of their CSAM use by loved ones or the legal system.

Several accounts reflected a strong internal conflict between desire and rejection of the behavior:

“I hate that I enjoy it and want to stop.”

This tension was accompanied by anticipatory fear of personal consequences:

“I always had a fear over my head that someday I’m going to lose everything.”

For some participants, distress extended beyond guilt and anxiety and was associated with more pervasive hopelessness and suicidal ideation.

“[My problem is that a] significant part of my thoughts is occupied by little girls, I can’t get rid of child porn, I constantly have suicide attempts…”

For some, the distress was closely linked to shame and perceived moral transgression.

“Probably the most embarrassing thing a person can do.”

Others attributed their negative emotions to the combined weight of punishment, stigma, and public exposure:

“You have to be honest and maybe the fear of punishment and everything else, the shame, the public stigma is the worst.”

Overall, negative emotions and distress were key aspects of CSAM use that directly affected participants in their daily lives.

##### 5.1.2.2 Lack of control

In some cases, there was significant anxiety about losing control completely and escalating to contact offending (even possibly offending against their own child), as well as the fear of being unable to stop their already occurring criminal behavior (such as CSAM use) altogether. These fears were described not only as abstract concerns but as recurring thoughts connected to everyday situations. Participants often reported heightened vigilance regarding their own reactions, particularly in situations involving children.

“One of the most important things, as I said, is my fear to harm someone in the future”

While some fears were oriented toward possible future harm, others were linked to immediate experiences of overpowering urges:

I get an urge, which completely takes over me. I am unable to stop myself. Which leads me to do things I don’t want.

For some participants, this anxiety was particularly salient in relation to their own children.

“And I’m a bit worried about how my body will react when my daughter grows up - on the other hand, I know that in reality I’m in control, but I would feel terrible if I got an involuntary erection in connection with her.”

Lack of control was therefore described both as a fear of future escalation and as a present-moment experience of urges perceived as difficult to regulate.

##### 5.1.2.3 Addiction-like narrative

Many participants described their relationship to CSAM as addiction-like in the sense that they were unable to control their behavior or suppress their desire to use CSAM despite its negative consequences. They linked their CSAM use to a cycle of highs and lows as if they were craving a drug: the initial rush, focus, or excitement they felt before or during use was soon followed by intense guilt, emotional withdrawal, and sometimes physical symptoms. Over time, the behavior became more compulsive and automatic. It gradually took over all aspects of daily life, leading to additional serious consequences.

“I experience it almost like a drug that makes me “disappear” from reality. I experience physical symptoms when I search for CSAM. Faster heart rate, enhanced focus, etc. Almost like a drug (but I do not do drugs). But the effects (disappearing from society, not contacting friends, not telling the truth about what I was doing), I would say, are similar to when people, for example, are at home drinking and being unavailable.”

For many, labeling their experience as an addiction seemed to express their internal conflict and the distress caused by engaging in behavior they felt incapable of stopping, despite its negative consequences.This could be seen as a mental aid in minimizing their own responsibility for their actions, which is discussed further in separate themes.*”It is scary how similar this is to the good old addiction”*.

Participants repeatedly used the term “addiction” to describe their experience of CSAM use. In their accounts, this comparison referred to feelings of craving, loss of control, and repeated engagement despite negative consequences. The addiction framing was used to describe the perceived compulsive and, in some cases, escalating character of their behavior, as well as the emotional highs and lows that accompanied it.

#### 5.1.3 Ego threat

Part of the problem of CSAM use or a sexual interest in children that participants articulated is that it threatens a person’s identity. The problem is so severe to them that fully acknowledging it affects the participants’ identity and self-image.

##### 5.1.3.1 Self-esteem reduction

One way in which participants described this identity threat was in relation to diminished self-esteem. Respondents described difficulties in maintaining a sense of self-worth in relation to recognizing the destructive nature of their behavior.

“I don’t feel like a good person because of it. It’s hard to love myself, it’s hard to feel proud of any success or anything good when, in the back of my mind, I know that something’s wrong with me, that I’m not okay.”

Participants also described difficulties integrating their sexual thoughts and behaviors with their existing sense of self, values, and identity. This incongruence often contributed to diminished self-worth and feelings of being internally divided. *“I often find these feelings difficult to process as they are at odds with my own ideology and identity.”*.

##### 5.1.3.2 Non-normality

Another dimension of ego threat was articulated through comparisons with “normality.” Participants evaluated themselves against their own understanding of what constitutes a “normal” person, particularly in relation to sexual urges and behavior.

“I’m a normal human being for 99% of my life. I go to work, I eat, I watch Netflix and I sleep. But because of what I did on that 1%, where I feel like I had no control over and feel terrible about. I always had a fear in my head that someday I’m going to lose everything.”

Participants often benchmarked themselves against their concept of a “normal” person, assessing whether their behavior was a minor deviation from an otherwise normal life or if the behavior fundamentally defined their personality. Others questioned whether such a distinction was sustainable:*”But I’m also kind of scared that that is just a lie I tell myself and who I really am is disgusting … who can never have a normal relationship.”*.

The concept of non-normality thus affected both participants’ internal sense of self and their expectations regarding acceptance, belonging, and the possibility of forming relationships with others.

##### 5.1.3.3 Rationalization of CSAM use and distancing to protect self-image

Potentially due to the threats to their identity, some participants did not fully acknowledge the seriousness of their CSAM use and instead distanced themselves from it in various ways in order to protect their self-image. For example, several participants minimized the seriousness of the fact that they were using child sexual abuse images or they convinced themselves that the society they live in is wrong about the harm that CSAM poses to children. This represents forms of offense-supportive cognitions. Consequently, many participants often feared being seen as pedophiles, either by their close ones or even by themselves, or argued pedophilia is just misunderstood in the society:*”I have the mental flexibility to convince myself that I’m not doing any harm by that since I technically do not influence anything on my own in this regard (I never pay for it or distribute it myself even)…”*.

Similarly, some framed the evaluation of their behavior as primarily a matter of societal judgment:*”Sometimes I wonder if I wouldn’t have the same worries if I was born a few decades earlier, or in a country where it was generally accepted.”*.

Several participants distanced themselves from the problem by attributing their behaviours to factors outside of their control. For instance, they claimed that they were simply “born this way” or that it was “just an addiction”. In their opinion, if their CSAM use was a disease, an “addiction,” there was hope for change. Framing it as an illness also allowed them to believe they were not inherently evil, a label they felt would otherwise be defined by their attraction. It could also be seen as a strategy to reduce responsibility, guilt, and shame. They had to constantly be on guard to avoid being exposed and they had to use euphemisms to hide their secret when trying to share it with others:”*It feels comforting and nice somehow, if it would be that [a disorder].”*.

Participants also frequently expressed this sense of relief through explicitly medical or brain-based language, describing their urges as symptoms, disorders, or neurobiological processes rather than purely intentional acts.

“Your brain tricks you to search for something illegal. Tricks you to believe that you need this material. It doesn’t tell you the guilt that you will feel after. In return you will feel forever in anxiety and fear of prosecution.”

Such descriptions framed the experience in physiological or neurological terms, emphasizing the role of the “brain” as an active agent in the behavior.*”I would say that: when I’m not feeling well and I’m in a dark part of life, my brain takes over me and tries to lead me down a path where I feel I have no control over what I do.”*

At the same time, some participants demonstrated awareness of these patterns and acknowledged their tendency to excuse or justify their behavior in ways that aligned with their self-image:*”I think I’m not too bad at finding excuses to suit my morality.”*

Across interviews, rationalization and distancing appeared in different forms, including minimization of harm, relativization of social norms, and medicalized explanations of behavior. These narratives coexisted with expressions of guilt, fear, and internal conflict, which were thematized before.

#### 5.1.4 Sensation seeking and compulsion

This theme captures a recurring pattern in which the CSAM use is described as driven by the pursuit of intense and highly stimulating experiences that cannot be found elsewhere. For some, the excitement stemmed not only from the sexual content itself, but also from its transgressive nature. Participants described “seeking the high”, a state they could not replicate through other forms of sexual or other activity. This cycle was often experienced as an automatic or compulsive pull, where unconscious triggers and urges culminated in active searching for CSAM.*”[It is] hard to get excited in real life from situations in real life. I use an easy way, online. (…) As time went on, normal porn didn’t excite me, so I started searching for more and more material.”*

In several accounts, the focus shifted from the content of the CSAM itself to the process of searching for it:*”I’m also often more turned on by the process of finding the porn than the porn itself.”*

Participants frequently described sensation seeking in connection with compulsive patterns of use and situational triggers. Across interviews, accounts reflected repeated attempts to control their behavior followed by renewed engagement in CSAM use, which they perceived as risky or problematic.

### 5.2 Perceived causes

#### 5.2.1 Innate versus learned

Participants’ relationship with CSAM was often strongly connected to how participants perceived the causes of their behavior. Many expressed a need to distinguish themselves from the “pedophile” identity, often emphasizing that “that is not me.” The way they described their sexual urges and CSAM use varied according to their cultural and family background, and reflected different explanatory frameworks: some referred to childhood events as the cause, others attributed their CSAM use to extreme stimuli seeking, “addiction”-type self-understanding, or attraction to younger individuals. Some participants reported an inability to identify the cause. Broadly, participants’ explanations clustered around three patterns: innate origin, learned or situational development, and uncertainty about causality.

##### 5.2.1.1 Innate

Some participants related their behaviour to the experience of an innate sexual preference which often brought negative feelings as they needed to acknowledge that they were born with different sexual urges than the majority population:*”I watch CSAM because I’m a pedophile, and it also helps me keep my behavior toward children under control”*Or:*” [It is something] I was born with (which is the most terrifying option for me) but it seems the most probable to me.”*

At the same time, even those who leaned toward an innate explanation of their CSAM use sometimes distinguished between attraction and action:*”It’s hard to say [what caused my problem], they say paraphilia is innate, I’m not sure if that also applies to paraphilia in action.”*

These accounts suggest that perceiving the interest in CSAM as innate did not necessarily eliminate a sense of agency, but it complicated how responsibility and control were understood.

##### 5.2.1.2 Learned

Other participants attributed their CSAM use to acquired psychosocial difficulties, both past and present. They described adverse childhood conditions (such as emotional insecurity or growing up in another country) as formative in the development of risky sexual behavior, including CSAM use. Other instances from their past included exposure to pornography or CSAM at a young age, or engaging with their sexuality very early on. Others cited emotional, physical or sexual abuse, either of them or a family member. Within this topic, we also identified a pattern where participants had sexual interest in children because they felt like they were still mentally or emotionally stuck at their age.

“I am almost certain that this is connected to the fact I was molested by my friends (2–3 years older than me) when I was like 9. I wouldn’t call it rape since as far as I remember I wasn’t actively against it, but the social hierarchy at that age is an external force on its own.”

Some participants referred to later life circumstances, such as retirement, loneliness or habitual pornography use, specifically in relation to the development or escalation of CSAM use behaviors rather than sexual attraction itself.*”I would like to say that I was watching too much porn and it turned into a bad habit, everything is so easily accessible.”*

In contrast, an upbringing characterized by education and stability was described as protective, offering both a moral framework and insight into what is right and wrong.An environment full of restriction was by comparison often seen as damaging. For example, some participants connected their interest in forbidden materials such as CSAM to restrictive attitudes towards pornography when growing up.

“Porn itself was forbidden. Both in my childhood home and in the current relationship. The forbidden has always been tempting.”

##### 5.2.1.3 Inability to identify cause

Sometimes participants were unable to speculate about the potential causes of their CSAM use. In these cases, participants described difficulty forming a coherent explanation of their behavior. When asked directly about possible origins, responses were often brief and marked by uncertainty.*”I don’t know why this is happening to me, or even if there really is a why”*

Such statements were expressed without further elaboration:*”In truth, I have no idea why I do it.”“*

For these participants, the absence of a clear explanation remained an unresolved aspect of their accounts.

### 5.3 Context, support and coping

#### 5.3.1 Unspeakable topic: the internalised taboo

This theme is characterised by tensions between suppression, hiding, and disclosure. Respondents described the need to hide and their struggle to share their CSAM use or sexual interest in children, and their difficulties describing their problem either to their close ones or to the interviewer. The problem was difficult to discuss for many reasons, the most prevalent reason was fear of getting caught by legal authorities (participants were aware of the illegality of their behavior) and the fear of losing their close ones if they learned about their behavior.*”In the worst-case scenario, the police show up at our house, which I wouldn’t exactly consider desirable.”*

Equally prominent was the fear of losing intimate relationships if their behavior was discovered:*”I’m afraid someone might catch me, like my girlfriend or friends … I’m afraid of losing them.”*

Another reason for the suppression was the lack of information or reception of contradictory information about the problem. We also observed that some participants with compulsive sexual behavior tended to distance themselves from the pedophile identity. They often expressed strong fear of being perceived as pedophiles - a label they perhaps associated with society’s view of being incurably ill and dangerous. “Addiction”, in their mind, could be cured, but pedophilia could not.*”And if in the end I find out that this (pedophilia) is what my sexuality just is, then I wouldn’t know what to do. I couldn’t accept this.”*

Most participants never told anyone about their CSAM use before the interview; others had only done so under coercive or clinical circumstances (an arrest or mandatory treatment). Even in these settings, disclosure was filled with anxiety and often expressed through euphemisms, vague language, or distancing from the topic.*”I would never voluntarily tell anyone about this.”*

One key aspect of hiding and disclosing was the tension between the two. The need to conceal the “unspeakable addiction” created a state of chronic vigilance and isolation. CSAM users live with a secret that feels impossible to share and has to be constantly hidden. However, once disclosure happens (whether voluntary or forced), they experience a paradoxical shift: a sense of relief or the beginning of a process through which the issue could be named, addressed, and managed. Yet, disclosure also brought real and immediate consequences - the fears that were once only hypothetical such as legal prosecution or family rejection suddenly become reality for some.

#### 5.3.2 Psycho-social regulators of use: a quality of life as a coping strategy

Relationships and overall well-being emerged as the central themes across nearly all interviews. When nurturing and fulfilling, the relationships and the individual’s well-being and psychological stability appeared to function as protective factors against the inclination to CSAM use. However, adverse circumstances in these areas increased the participants’ likelihood of CSAM use.

It is important to point out that neither relationships or well-being was depicted as an absolute cause or a definitive solution. Rather, both positive and negative experiences in their lives served as influential elements within the complex interplay of psychological and behavioral dynamics. In this sense, protective factors did not eliminate the behavior but appeared to strengthen participants’capacity to manage, resist or disengage from CSAM – at least for a while.

We could categorize this effect as the influence of quality of life, where it may play a regulatory role. E.g., in some of the participants, the less they reported adverse life circumstances (such as frequent negative moods and emotions, and the subsequent inability to cope with them, stress, lack of relationships and loneliness, etc.), the less likely they were to use CSAM:*”it gets worse when I am tired or stressed or procrastinating or simply when I am alone for a long time”*.

Cultural concepts such as morals and values (such as, “Hurting children is wrong” or “Children should be protected”) were another example of psychosocial regulators of CSAM use. A significant number of the participants experienced the contradiction between their needs and values. While the existence of moral values mostly contributed to their inner conflict, their moral competence as the ability to stick to one’s principles acted as a motivator to help stop the participant’s CSAM use.

“My conscience helps me to resist those urges; The most important thing, in my opinion, is the principles of ethical behaviour, generally valid in our country and culture.”

“Even if I got away with it [if the police never found out], I’d still have a problem with myself.”

These regulatory mechanisms were not static but interacted with longer-term psychosocial conditions. Long-term social stressors, such as chronic loneliness, unstable or absent relationships, and ongoing difficulties in coping with emotions, emerged as important background factors influencing CSAM use. In addition to these social stressors, boredom was also described as a separate trigger, particularly when combined with a lack of meaningful activities or social engagement. Participants who struggled to manage these factors often described being left without adequate emotional regulation strategies or social support. This lack of coping resources, in combination with easy access to CSAM, increased the likelihood of using such materials.

“I’ve only done it when I’ve been incredibly lonely, even when I’ve had nothing to do because I was off work. [ … ] Yes, but I also need to feel bad, because sometimes I have been bored but found other constructive things to do. But loneliness is contributing. For example, the holidays, when many friends are away. “

Beyond relational and emotional circumstances, participants also described internal value systems as important regulators of CSAM use. Current stable psychosocial conditions appeared to mitigate CSAM use. Even in cases where participants were currently struggling, they recalled that when they were doing better, the need for CSAM consumption subsided. *“In periods when I feel good and have enough energy, I have no problem not thinking about CSAM at all and being functional.”*

Overall, CSAM use was described as sensitive to fluctuations in psychosocial stability, emotional regulation, and moral self-concept. Improvements in these areas appeared to reduce vulnerability, whereas their deterioration increased it. Importantly, these protective or regulatory effects were not uniform across participants and they were not present or mentioned by all of them. While some participants described a reduction in the intensity of frequency of CSAM use during periods of improved psychosocial stability, others did not experience these to the same extent. Rather than leading to a cessation of behavior, those factors appear to modulate it in varying ways. The participants who were able to identify and reflect on some of these influences were also the ones to describe at least a temporary reduction in use or an improved ability to resist their urges for a while.

#### 5.3.3 Stigma and social exclusion: shame, silence, and the loss of connection

This theme examines the experienced stigma in the context of social relationships. In this case, either to close people or clinicians. Stigma, associated with guilt and shame, directly impacted the participants’ willingness to share their problem with others and in more severe cases, this stigma was internalized and progressed into self-hatred, affecting their ability to form and partake in relationships in general.*”I might have a friend I could talk to, but not like I feel I can go into the depth of the problem”*.

This hesitation illustrates how stigma constrained not only disclosure but also the depth and quality of interpersonal connection.*”[Are there any kinds of support that make your problems (CSAM-use) better, such as support from family, friends, or others]?*

*No”*.

Such responses reflect the extent to which stigma limited access to perceived social support, even when supportive figures were potentially available. As a result of the stigma, some participants were hesitant to share, or decided not to share any information regarding their problem with people who would normally provide support.

Some participants who shared their problem with others struggled with negative consequences as a result, such as permanent loss of the relationship. Experiences like this, or the fear of experiences like this, lead participants to rely mainly on themselves when seeking support, often isolating them further. For some, prolonged silence and concealment contributed to deeper internal distress.*”This is one of those questions that tears me up inside, and not understanding where the dark part is coming from “*.

Participants often described being left alone with their sexual experiences and feelings throughout life. Feelings of shame were central to this isolation, reinforcing the reluctance to share their experiences. Support from close ones was only limited, with more substantial help appearing only after being caught or through contact with a specialized caregiver. Many reported thinking about the problem but not understanding it, and experiencing a lack of support in making sense of it.*”I wouldn’t say anything. In fact I was so afraid and ashamed of myself that I no longer talk to anyone about anything outside my immediate family.”*

Overall, experienced and internalized stigma appeared to reinforce silence, restrict supportive connections, and intensify psychological isolation. Rather than facilitating help-seeking, stigma often entrenched self-reliance and social withdrawal.

## 6 Discussion

In this paper, the Cultural Formulation Interview method was adapted for use in a chat-based interview format for the first time.This enabled meaningful dialogue with European respondents while maintaining anonymity and reducing barriers to participation among CSAM users. This population includes both the non-forensic population (many of whom are active on the Dark Web) and the forensic population under criminal investigation for CSAM use. This study is distinctive in its person-centred approach, yet it remains firmly grounded in the primary objective of child protection. By analyzing the psychological drivers of those who consume child exploitation material, the project aims to inform more effective prevention of child sexual abuse and the proliferation of CSAM. Despite the high levels of stigma (26, 86) associated with CSAM use, accessing this difficult-to-reach group is essential for identifying help-seeking needs of CSAM users (12, 26) and preventing future harm. Beyond providing a thematic analysis, this study advances the understanding of CSAM use by situating participants’ narratives within stigma-related, motivational, cognitive, and epistemological frameworks. This integrative approach enables a nuanced interpretation of how internal conflict, meaning-making, and regulation are shaped by social context, stigma, and perceived responsibility, ultimately serving the broader goal of victimization prevention.

### 6.1 Perception of the problem: identity, conflict, and the dynamics of fear

The findings demonstrate that CSAM use profoundly disrupts individuals’ perceptions of themselves, tand the problem itself.

When analyzing participants’ lived experience, it is important to distinguish between two separate internalized concepts: the internalization of societal views regarding a pedophilic sexual preference, and the internalization of views regarding the criminal behavior of CSAM consumption. While some participants struggled with the identity as someone who is attracted to children, others struggled with the concept of consuming CSAM – one fear could be present without the other, but oftentimes, participants were afraid of both. Both of these fears result in chronic shame, self-monitoring, and anticipatory fear of social exclusion (58, 59, 87). This internalized stigma constitutes a core backdrop against which all themes are articulated. In Nordenfelt and Twaddle’s (75) terms, these experiences are best situated at the interface of *illness* (subjective distress) and *sickness* (socially imposed moral meanings), rather than within a disease-based framework.

These inner conflicts manifest in the data on two psychological levels: struggles over action (“doing”), i.e. how individuals navigate their immediate behavioral choices, and struggles over personhood (“being”), i.e. how individuals navigate their global identity as consumers of this material.

Within the theme *CSAM as a Conflict*, this struggle is localized at the action level. The intense internal friction is expressed through a desire for help and to stop using CSAM, yet their narratives reveal offense-supportive cognitions. These cognitions likely serve a regulatory function, reducing feelings of shame and temporarily neutralizing moral inhibitions and enabling continued CSAM use despite the individual’s underlying distress. For example, emphasizing compulsion, inevitability, or temporary loss of control. The presence of such cognitions has been widely noted elsewhere (63–66, 88). These problematic cognitions are consistent with cognitive-motivational models as they appear to function as strategies for managing dissonance between desire and moral standards (73).

When this behavioral struggle becomes deeply internalized, it transitions into an identity crisis, as explored in the theme *Ego threat*. Individuals discuss their struggle with finding themselves fundamentally unable to reconcile the severe harm they acknowledge causing with their core self-concept. This identity-level distress is heavily bounded by the surrounding cultural background; participants exhibit an acute fear of social and legal condemnation and a profound anxiety regarding the consequences of violating the shared moral frameworks of their community.

Motivational frameworks further differentiate these themes. From a motivation-facilitation model perspective, both struggles involve persistent sexual motivation interacting with strong inhibitory forces (62). In *CSAM as a Conflict*, this interaction is experienced dynamically at the level of behavioral regulation, marked by struggle and oscillation between wanting to stop and failing to do so. In *Ego Threat*, it is experienced more globally and relatively statically, as a threat to identity coherence and self-worth. Whereas the former emphasizes ongoing conflict, the latter reflects erosion or collapse of self-evaluative stability.

The overwhelming atmosphere of distress is further elaborated in the theme *Lived Experience of Fear*. Fear is identified as a defining, multi-layered emotion that structures the entire consumption experience. Critically, the negative emotions associated with CSAM use construct a psychological paradox: they simultaneously drive the desire to stop and paralyze the capacity to do so. Some participants describe a cyclical trap of feeling profoundly negative about their consumption, experiencing acute shame over their inability to stop, and facing escalating anxiety regarding their perceived addiction and potential behavior escalation. Similar themes have been described in literature before: addiction-like narratives are a commonly observed theme in the research (10, 11, 63, 66) and the cyclical nature of this experience has been acknowledged by Meridian et al. (65) in their case formulation model of pathways to CSAM offending.

Based on these findings, several practical implications for prevention and intervention emerge. Interventions may build on the emerging moral awareness evident in participants’ narratives, using it as a lever for behavioral change while supporting responsibility without reinforcing global self-condemnation. Given the central role of overwhelming fear and shame, professionals should be prepared to work explicitly with emotion regulation and the processing of distress associated with CSAM use. Brief, structured online interventions may particularly benefit from Motivational Interviewing to address ambivalence and enhance readiness for change (89, 90), alongside cognitive-behavioral and relapse prevention techniques targeting trigger identification, affect regulation, and cognitive restructuring (91, 92). In contrast, deeper identity-level work with chronic shame, internalized stigma, and self-concept repair may require longer-term therapeutic engagement, as unprocessed shame has been associated with avoidance, secrecy, and relapse risk (93). While this psychological profile may not apply universally to all users, our data suggest it represents a prevalent clinical reality that warrants structured, shame-sensitive intervention approaches.

### 6.2 Perceived causes: innate versus learned and the heterogeneity of motivations

When addressing the etiology of their CSAM use, participants’ narratives mirror a broader scientific reality: the precise causal pathway remains fundamentally ambiguous, presenting as innate, learned, or occasionally both. Rather than yielding objective etiological data, discussions of causality function primarily as sites of active meaning-making.

A central feature of this theme is identity distancing. Many participants explicitly rejected identification with the label “pedophile,” emphasizing that “this is not me,” even when acknowledging attraction to younger individuals, or their engagement with CSAM. This distancing could be understood through stigma-related frameworks as a response to their experience of internalizing stigma, where the social meaning attached to pedophilia is perceived as incompatible with an acceptable self-concept (58, 59). CSAM users’ causal explanations may thus serve a protective function, allowing participants to attribute behavior to external or developmental factors rather than to a fixed, morally condemned and ego-integrated identity.Interestingly, the identity distancing attempts reveal a clear potential for individuals to engage in the overexplaining of their issues. Drawing on dual-process cognitive theories, these explanations can be understood as conscious, deliberative attempts to reinterpret automatic sexual responses in ways that remain compatible with personal and social values and self-concept (73). These findings align also with Knack et al. (66), Merdian et al. (2011, 65 and Quayle and Taylor (63), who acknowledge the etiological factors behind CSAM use often act as justifications for CSAM users’ criminal interests.

Importantly though, uncertainty itself (expressed as an inability to identify causes and avoidance) emerged as a distinct experiential position, reflecting both limited access to internal processes and the burden of navigating causality under conditions of shame and fear.

Overall, *Innate versus Learned* highlights how perceived causality functions as a key site where responsibility, identity, and stigma concepts intersect. Rather than revealing stable etiological beliefs, participants’ accounts demonstrate flexible and context-sensitive meaning-making strategies that allow them to navigate their accountability while resisting totalizing moral judgments and self-concept.

The nature of the identity struggle carries important implications for public outreach and primary prevention. If campaigns are framed exclusively around a “pedophile” identity, they will likely fail to engage many active CSAM users. Because many users operate in deep psychological denial regarding a pedophilic identity, identity-focused messaging drives concealment. Public prevention strategies should thus shift their vocabulary away from identity labels and focus instead on behavioral markers, such as “concerning pornography use” or “out-of-control digital behavior.”

At the same time, practitioners must be prepared to engage individuals presenting with compulsive sexual use patterns without forcing labels the individual does not identify with. While the identity as a pedophile proves to be an important part of the core identity of some CSAM users, short-term interventions should rather minimize focus on definitive etiological origins and instead prioritize present-moment behavioral regulation and accountability, leaving the long-term therapeutic focus on self-acceptance of the “pedophile” identity a goal of long-term therapeutic programs (e.g. BEDIT, 94). The results also highlights that claims about congenitality, immutability and non-treatability of sexual preference create powerful narratives in public space that are then difficult to dispel and could, under certain circumstances, have negative effects on motivation to seek help and behavioral change (95–97). The wider than black and white concept of the treatment goals and outcomes need to be promoted and explained in public presentations and specified in the treatment goals.

### 6.3 Context and support: coping, isolation, and the space of the online world

The relational and contextual dimensions of CSAM use are best captured across the themes of the *Unspeakable Topic: The Internalised Taboo*, *Psycho-Social Regulators of Use – A Unique Life-Coping Strategy*, and *Stigma and Social Exclusion*. Our findings indicate that CSAM use is frequently used as a maladaptive mechanism to cope with acute emotional distress. This aligns with foundational perspectives by Quayle (63) regarding individuals who were not equipped with adaptive emotional processing skills by their primary caregivers, as well as frameworks by Knack et al. (66) emphasizing the powerful desire to avoid or numb emotional pain, and Merdian et al. (65) who argues that emotional dysregulation proves to be one of the more common vulnerabilities towards CSAM use.

When individuals perceive themselves as fundamentally “wrong” or broken due to their desires or behaviors, they experience a collapse in interpersonal trust. This leads to profound social isolation. In line with stigma-related stress models, anticipated rejection and identity concealment emerged as dominant strategies, resulting in chronic vigilance and emotional isolation (58, 59). This isolation severely restricts CSAM users’ access to prosocial, adaptive coping strategies. The dynamic highlights the deep cultural significance of support structures whereby the resources an individual needs to change are intrinsically embedded within the culture they inhabit.

*While Unspeakable Topic* captures the communicative barrier itself, *Stigma and Social Exclusion* highlights its interpersonal consequences. Participants’ accounts reveal that stigma was not only anticipated but often internalized, progressing into self-hatred and withdrawal from relationships. In some cases, participants reported that even when potential sources of support existed, the perceived risk of disclosure outweighed any anticipated benefit, which is in line with Elchuk et al. (58). These experiences underscore how social exclusion operates not only through overt rejection but through the internal regulation of behavior and emotion.

Conversely, when the aftermath of stigma triggers total social withdrawal from the offline world, individuals experience a dangerous displacement. They migrate deeper into online environments where, as noted by Merdian et al. (64, 65, the virtual space stands ready to provide immediate reinforcement for their behavior. Social isolation thus acts as a primary contextual regulator, actively driving the transition from offline distress to online escalation.

In contrast, *Psycho-Social Regulators of Use* foregrounds the mitigating potential of relational and contextual stability. Participants described periods of improved emotional well-being, meaningful relationships, structured daily routines, and social connection as protective, often coinciding with reduced CSAM use, consistent with Merdian et al. (65). Conversely, loneliness, boredom, depression, and lack of meaningful activity were repeatedly identified as triggers of CSAM use. This pattern aligns with motivational and facilitation-based models, in which stressors and affective states interact with sexual motivation to influence behavior (62). Importantly, these accounts emphasize regulation rather than etiology: support does not eliminate sexual interest but appears to strengthen inhibitory and coping capacities.

From a clinical perspective, these findings suggest that low-threshold online interventions may be particularly valuable in addressing stigma-related silence before more intensive therapeutic work becomes possible. Consistent with research on help-seeking barriers in minor-attracted and CSAM-using populations (26, 86a; 12), brief interventions incorporating psychoeducation, motivational interviewing, and supportive guidance may facilitate initial disclosure and engagement. Longer-term therapeutic work may then focus on reducing internalized shame, strengthening interpersonal functioning, and rebuilding prosocial sources of support.

Taken together, the themes within *Context and Support* demonstrate that CSAM-related distress and regulation cannot be understood in isolation from the sociocultural environment. Silence, stigma, and exclusion constrain meaning-making and help-seeking, while relational stability and psychosocial resources help reduce risk. These findings underscore the need for low-threshold, confidential, and non-judgmental support options that address stigma-related silence as a primary barrier to help-seeking. Creating spaces where experiences can be articulated without immediate moral or legal collapse may be a necessary precondition for effective prevention and sustained behavioral regulation.

### 6.4 Interconnected themes in lived experience

Although the distinction between *Perception and Description of the Problem* and *Perceived Causes* and *Context and Support* is analytically useful and determined by the structure of the CFI tool, participants’ narratives indicate that these themes are closely intertwined in lived experience. Internal conflict, ego threat, causal attributions, and stigma-related silence frequently co-occurred, suggesting that meaning-making, responsibility negotiation, and regulation unfold simultaneously rather than completely separate dimensions. As a result, certain experiences contribute meaningfully to more than one theme, which reflects their multi-layered character. This overlap thus reflects the nature of the problem itself and is consistent with approaches that highlight concepts as interconnected processes rather than discrete categories (59, 98). Attempts to fully disentangle these experiences into clearly bounded categories would risk oversimplifying the phenomenon as themes in reflexive thematic analysis are understood as overlapping and interpretative patterns of meaning (81). Separating the elements would thus likely come at the cost of losing the dynamic interplay that characterizes participants’ lived experience.

### 6.5 CFI: Impact of culture

Although the present study utilized the Cultural Formulation Interview (CFI), the aim was not to conduct a systematic comparison between national cultural groups. The nature of the sample, the uneven distribution of participants across language groups, and the predominance of English-language interviews did not allow for robust cross-cultural comparisons at the country level. Instead, the CFI was used as a framework for exploring how participants understood and articulated their subjective experiences within broader sociocultural and moral contexts.

Across interviews, participants’ accounts reflected a largely shared European sociocultural environment characterized by strong moral condemnation of CSAM use, emphasis on individual responsibility, child protection values, and fear of legal and social consequences. While some interesting differences between language groups were observed (for example, greater references to psychoeducation and mental health literacy in parts of the Swedish sample), these patterns were not sufficiently systematic to support comparative interpretation. The present analysis therefore focused primarily on commonalities across participants rather than on contrasts between countries or language groups.

In this context, culture should not be understood narrowly as nationality or ethnicity, but more broadly as a shared system of meanings, moral expectations, legal norms, and socially embedded understandings of sexuality, responsibility, and deviance. The results suggest that these culturally embedded frameworks represented an important internalized context within which participants experienced shame, secrecy, self-evaluation, and help-seeking. The findings in sum indicate that participants were navigating their subjective experiences within sociocultural contexts in which CSAM use is strongly stigmatized and socially unacceptable.

At the same time, it has to be acknowledged the sample consisted of individuals actively seeking help, therefore internalized values and moral concerns in the present data cannot be assumed to represent all CSAM users.

### 6.6 Practical implications for therapeutic and preventive interventions

The findings suggest that interventions for CSAM users should explicitly acknowledge internal conflict, ambivalence, and moral concern rather than assuming denial, lack of insight, or callousness. Across domains, most participants demonstrated awareness of harm and distress related to their behaviour, yet this awareness did not automatically translate into behavioural change. Interventions may therefore benefit less from increasing awareness of consequences and more from strengthening self-regulation and translating existing motivation into sustained behavioural change. In low-threshold and online settings, Motivational Interviewing may be particularly useful for addressing ambivalence and facilitating engagement (89), while the addiction-like narratives described by many participants further support the use of cognitive-behavioural and relapse-prevention approaches targeting trigger identification, affect regulation, cognitive restructuring, and the development of alternative coping strategies (92, 93). The theme *Innate versus Learned: Balancing on the Border of Causality and Responsibility* further suggests that therapeutic work should help individuals tolerate uncertainty regarding causation while maintaining responsibility for behaviour, thereby reducing defensive explanations and facilitating engagement in change-oriented work. Participants rarely described reductions in CSAM use as resulting from changes in sexual interests; instead, improvements were more often associated with greater social connectedness, meaningful relationships, daily structure, and emotional stability, highlighting the importance of addressing loneliness, emotional dysregulation, and psychosocial functioning alongside behaviour itself. This finding suggests that effective prevention may depend as much on strengthening protective psychosocial resources as on reducing problematic behaviour itself.

Internalized stigma and ego threat emerged as prominent themes and appeared closely intertwined with shame, secrecy, and social withdrawal. Consistent with previous findings that internalized pedonegativity may fuel concealment and disengagement from support (87, 99), the present findings suggest that interventions should aim to reduce shame-based disengagement while preserving ethical accountability. This approach is reflected in existing prevention initiatives such as ReDirection, Inform Plus, and Engage Plus (29) and Troubled Desire (15), where stigmatizing language is deliberately avoided. Similarly, Czechia’s Project Paraphile frames its prevention message around the principle “*You are responsible for your actions, not your thoughts*” (100), while Germany’s prevention network Dunkelfeld combines a clear stance against offending with accessible pathways to support (25).

These findings further suggest that prevention campaigns should avoid essentialist messages implying that individuals are inherently “bad” or beyond help, as such messages may reinforce shame, secrecy, and avoidance of support (26, 59, 98, 101, 102). Instead, campaigns should communicate both the harmfulness and illegality of CSAM use and the availability of confidential support before behavioural escalation occurs. Finally, while anonymous low-treshold online interventions may be particularly effective in reducing barriers to disclosure and providing an entry point into care for undetected individuals (59, 101), the present findings also highlight their limits. For individuals experiencing chronic shame, profound loneliness, identity-related distress, or broader psychosocial difficulties, longer-term face-to-face therapy may be required to address entrenched interpersonal and emotional vulnerabilities and support sustainable behavioural change (65, 98).

At the same time, the findings highlight the importance of culturally and structurally informed prevention strategies. Participants’ experiences of guilt, stigma, disclosure, and help-seeking appeared embedded within broader social, legal, and healthcare systems. Across Europe, prevention infrastructures differ in the availability of specialized services, confidentiality regulations, reporting frameworks, and pathways to care, all of which may influence engagement with prevention and treatment services (13, 28, 33). Cultural adaptation should therefore extend beyond language translation and include sensitivity to local understandings of responsibility, confidentiality, and acceptable disclosure. In this context, the Cultural Formulation Interview proved valuable for capturing these nuances and may provide a useful framework for adapting preventive interventions across different national contexts.

### 6.7 Limitations

Several limitations of the present study should be acknowledged. Some of these are inherent to qualitative research and were addressed through methodological safeguards. To mitigate potential researcher bias and enhance analytic reliability, coding was conducted in teams and dyads, supported by regular reliability checks and shared discussions across coding groups to ensure consistent understanding of the coding framework.

We cannot rule out inaccurate responses from participants, or answers influenced by social desirability bias. However, given the extreme stigmatization of CSAM use documented both in this study and in prior research (59, 99, 101), the anonymity afforded by the Bridge project was considered a necessary condition for participation and trust. Due to this anonymity, follow-up interviews to clarify meanings or verify responses were not possible, creating an important trade-off between data depth and participant safety. In addition, the sample comprised individuals who were already seeking help which may limit transferability: compared with people who use CSAM but do not seek support, participants may be more self-reflective, more likely to problematize their behaviour, and more oriented toward change, potentially influencing both what they disclosed and how they framed their experiences.

Checking with participants to validate the interpretation of the themes was not possible due to the anonymous design of the project, which precluded any form of re-contact with respondents after their participation in the project ended. To partially offset this limitation, the thematic interpretation was reviewed by an independent qualitative methods expert (G.B.) and subject to ongoing consultation with the Czech principal investigator (K.K.) throughout the analytic process.

Finally, the use of the CFI itself entails certain limitations. Both researchers and participants noted overlap between domains, reflecting the pervasive and multifaceted nature of CSAM-related distress.

### 6.8 Ethical reflections

The interpretation of the data was conducted under the strict oversight and approval of the Swedish Ethical Review Authority (Dossier No. 2023-02321-01) and by local ethics committees, ensuring that the study remained within rigorous ethical boundaries even when dealing with highly sensitive content. The language utilized throughout the results and discussion sections often mirrors the specific terminology and narrative found within the user data. To effectively shed light on the inner workings and offence supported cognitions of those who watch this material, we must analyze their narratives in their original context. However, the adoption of this language for analytical purposes in no way implies an endorsement of the subjects’ views or a validation of their behaviors. Instead, it serves as a necessary tool to deconstruct the harmful ideologies that sustain them.

### 6.9 Future research

Future research should extend the present findings by exploring additional interpretative frameworks that may further illuminate the internal conflicts and meaning-making processes identified in CSAM users’ narratives. One promising avenue involves secondary qualitative analyses of existing and future datasets using psychodynamic or structural models of personality to examine how internal conflict, ego threat, and moral self-evaluation are organized and negotiated. Although such models were not applied in the present analysis, they have previously been proposed as useful for understanding inner conflict and regulation in sexual offending populations (20). Applied as interpretative rather than diagnostic frameworks, these approaches could offer complementary insights into the psychological dynamics underlying ambivalence, responsibility, and self-concept without contradicting the current stigma- and motivation-based interpretation.

In parallel, future research should differentiate between subgroups within the heterogeneous population of CSAM users, with particular attention to individuals at higher risk of escalation or contact offending. Prior studies indicate substantial variability among CSAM users in terms of sexual interests, antisociality, self-regulation, and risk trajectories (19, 62, 68). Comparative analyses between lower-risk and higher-risk groups could clarify how internal conflict, stigma, causal attributions, and help-seeking differ across risk profiles and how these differences should inform patient-centered prevention and intervention strategies.

A further key direction concerns the systematic examination of sociocultural and structural influences on CSAM-related meaning-making and intervention engagement. While the present study included participants from multiple European countries, the data did not allow for cross-national comparisons. Future research would should include a wider range of European states to examine how differences in legal frameworks, mandatory reporting obligations, criminal justice approaches, availability of preventive services, and public discourse shape client-centered perspectives and culturally adjusted intervention strategies (22, 59, 86, 98). Extending such comparisons beyond Europe would further enable analysis of how broader sociocultural value systems and legal traditions influence stigma, disclosure, and engagement with online interventions. Given evidence that digital, low-threshold interventions can reduce barriers for highly stigmatized populations (12, 26, 86), future studies should specifically investigate how culturally embedded norms and legal contexts shape the acceptability and effectiveness of online, patient-centered prevention strategies.

## 5 Conclusions

This study demonstrates the value of adapting the CFI to an anonymous, chat-based format for accessing the lived experiences of CSAM users, including individuals unlikely to engage with traditional research or clinical services. To our knowledge, this is the first study to apply the CFI in an online, text-based setting with this highly stigmatized and hard-to-reach population, including users active in surface and dark web environments. This methodological innovation enabled culturally informed, patient-centred data collection while reducing barriers related to fear of disclosure, legal consequences, and social judgment.

The findings reveal CSAM use as a heterogeneous and deeply distressing experience shaped by internal conflict, identity threat, perceived loss of control, and pervasive stigma. Interpreted through stigma-related, motivational, cognitive, and illness-sickness frameworks, participants’ narratives indicate that explanations of causality and responsibility function primarily as meaning-making strategies for negotiating shame, agency, and self-concept rather than as stable etiological beliefs. Across themes, secrecy, social isolation, and limited access to support emerged as central constraints, while relational stability, emotional well-being, and life structure functioned as key contextual regulators. Notably, participants rarely associated reductions in CSAM use with changes in sexual interests themselves, but rather with improvements in social connectedness, emotional stability, and broader psychosocial functioning.

By integrating a culturally informed qualitative methodology with contemporary theoretical models, this study advances CSAM research beyond diagnostic and risk-focused perspectives toward a nuanced understanding of lived experience. The findings underscore the importance of accessible, low-threshold prevention services that can engage individuals before behavioural escalation occurs, while also highlighting the need for longer-term interventions addressing shame, social isolation, and broader psychosocial vulnerabilities. More broadly, the study contributes to the development of culturally informed, patient-centred prevention strategies that complement law-enforcement approaches by reaching individuals who might otherwise remain hidden from existing systems of support.

## Data Availability

The datasets presented in this article are not readily available because consent from the participants was not given to share the data publicly nor upon request.Requests to access the datasets should be directed to Mgr. Kateřina Klapilová, PhD, katerina.klapilova@nudz.cz.

## Footnotes

1 Although the CFI is recommended for initial assessments with all patients, it is especially valuable when the patient and clinician differ in social or cultural background (American Psychiatric Association 2022). For this analysis, European culture is treated as a single overarching framework. We focus on themes common across Europe, while acknowledging their varied expression in specific regions. Readers should keep this in mind when applying the model in practice; country-level cultural nuances, where available, are addressed separately.
