# Supplementary material for: Identity under threat: internal conflict and stigma in undetected child sexual abuse material users
Source: Front Psychiatry. 2026 Jul 31;17:1830667. doi: 10.3389/fpsyt.2026.1830667 (PMC13473276; doi:10.3389/fpsyt.2026.1830667)
Supplement: Supplementary file 1 [file DataSheet1.pdf]

The Cultural Formulation Interview (CFI)

Modified Structure for BRIDGE Practitioners

Content

HOW TO WORK WITH THIS DOCUMENT? 2

INTRODUCTION 2

1. DOMAIN: CULTURAL DEFINITION OF THE PROBLEM 2

2. DOMAIN: CULTURAL PERCEPTIONS OF CAUSE, CONTEXT, AND SUPPORT 3

2.1 CAUSES 3
2.2 STRESSORS AND SUPPORTS 4
2.3 CULTURAL IDENTITY 5

3 DOMAIN: CULTURAL FACTORS AFFECTING SELF-COPING AND PAST HELP-SEEKING 5

3.1 SELF-COPING 5
3.2 PAST HELP-SEEKING 5
3.3 BARRIERS 6

4 DOMAIN: CULTURAL FACTORS AFFECTING CURRENT HELP-SEEKING 6

4.1PREFERENCES 6
4.2 CLINICIAN-PATIENT RELATIONSHIP 6

CONCLUSIONS 7

HOW TO WORK WITH THIS DOCUMENT?

BLACK TEXT *- to be sent to the participant (16 CFI core questions; follow-up information for participants)*

GREEN TEXT *- Alternative wording of core CFI questions, which are marked as **AQ.***

BLUE TEXT *- Supplementary questions (marked as **SQ**) extending the core CFI questions.*

RED TEXT *- Prompts and instructions to follow.*

INTRODUCTION

Hello, I'm online. Please drop me a message when you join the chat.

OR

I see you've logged into the chat. Can we get started?

[the participant's answer]

I want to start by giving you some basic information about today's interview, but first, I would like to make sure that you have given informed consent during the screening interview to participate in this CFI interview, can you confirm that for me?

[the participant's answer]

Thank you for the confirmation. The interview aims to get a better understanding of the problems that bring you here so that I can help you more effectively. I want to know about your experience and ideas. I will ask some questions about what is going on and how you are dealing with it. Please remember there are no right or wrong answers. The interview is estimated to last 60 minutes. I also ask you not to share any personal information during the interview that would reveal your identity.

[the participant's answer]

So, to begin, I'd like to know more about how you found out about us. Try to remember and describe your journey from the moment you found out about us to this interview.

1. DOMAIN: CULTURAL DEFINITION OF THE PROBLEM

1. What brings you here today?

People often understand their problems in their own way, which may be similar to or different from how doctors describe the problem. How would you describe your problem?

AQ (instead of 1. core question)

I want to know more about the concern that caused you to seek our research project. I'm interested in your perspective and your way of describing it. Can you describe what led you to do this, what concerns or what the problem was?

AQ (in case he/she has a problem formulate his/her concerns)

I remember that you mentioned during the last interview that you are concerned about [formulation from screening interview], is it okay if I use this formulation in the following question?

OR

SQ2:

What did you know about your [PROBLEM] before it affected you?

SQ5:

Have you seen on television, heard on the radio, read in a magazine, or found on the internet anything about your [PROBLEM]? Please tell me more about it.

2. Sometimes people have different ways of describing their problem to their family, friends, or others in their community. How would you describe your problem to them?

AQ (in case he/she didn't disclose his/her problem to anyone):

I understand this is not easy to talk to someone about. In this case, try to imagine that I am someone close to you, a relative, a friend, or a colleague, and try to explain your problem to me. How would you describe your problem?

AQ (in case he/she didn't disclose his/her problem to anyone):

I understand that this is not easy to talk to someone about. In this case, try to imagine a situation where you are trying to explain your problem to your family, friends, colleagues, or partner. How would you describe it to them?

3. What troubles you most about your problem?

SQ1:

How has your [PROBLEM] affected your ability to do the things you need to each day, that is, your daily activities and responsibilities?

This is the end of the questions from the 1st domain, the following questions are about the 2nd domain, which focuses on the perception of the cause and context of your problem and the sources of support in your life.

2. DOMAIN: CULTURAL PERCEPTIONS OF CAUSE, CONTEXT, AND SUPPORT

2.1 CAUSES

4. Why do you think this is happening to you? What do you think are the causes of your [PROBLEM]?

SQ11:

Do you consider your [PROBLEM] to be serious? If so, why? What is the worst that could happen?

PROMPT FURTHER IF REQUIRED:

Some people may explain their problem as the result of bad things that happen in their life, problems with others, a physical illness, a spiritual reason, or many other causes.

*Before asking the following questions, make sure you use the terms used by the client to describe their problem in Domain 1 as a substitute for the term "PROBLEM." (e.g., In some cases, the client may not perceive their paraphilic preference as problematic but may believe that the problem is that

the paraphilia may pose a risk of harm to the child. In that case, we are not interested in the causes of the paraphilic preference. We are still interested in the causes of the paraphilic preference posing a risk.)

*As a possible solution, we recommend agreeing on the problem formulation with the participant before asking the following questions. You can also use this solution if the client is using an inappropriate term to describe their problem (e.g., "child pornography") and you find it problematic to use this term.

5. What do others in your family, your friends, or others in your community think is causing your [PROBLEM]?

AQ (in case he/she didn't disclose his/her problem to anyone):

Thank you for pointing out that you have yet to share this with anyone. In this case, think about how members of your social network would interpret the fact that you have this problem. What would they say is causing your problem?

AQ (in case he/she didn't disclose his/her problem to anyone):

Thank you for pointing out that you have yet to share this with anyone. Hypothetically if members of your social network know about your problem, what would they think was causing your problem? What would they say is causing your problem?

SQ5:

Are there people who do not know about your [PROBLEM]? Why do they not know about your [PROBLEM?]

2.2 STRESSORS AND SUPPORTS

6. Are there any kinds of support that make your [PROBLEM] better, such as support from family, friends, or others?

→AQ:

…support from peer groups, self-help books, etc.

7. Are there any kinds of stresses that make your [PROBLEM] worse, such as difficulties with money, or family problems?

SQ7:

Have you experienced discrimination or been treated badly as a result of your background or identity? By background or identity, I mean, for example, the communities you belong to, the languages you speak, where you or your family are from, your racial or ethnic background, your gender or sexual orientation, and your friend or religion. Have these experiences had an impact on [STRESSORS] or your [PROBLEM]?

In the middle of this interview, before I proceed to the next question, I'd like to inquire whether you'd like to take a 3-minute break or if you'd instead continue the interview uninterrupted. What would you prefer?

2.3 CULTURAL IDENTITY

*If you have asked supplementary question SQ7, skip following prompt:

Sometimes, aspects of people's background or identity can make their [PROBLEM] better or worse. By *background* or *identity,* I mean, for example, the communities you belong to, the languages you speak, where you or your family are from, your race or ethnic background, your gender or sexual orientation, or your faith or religion.

8. For you, what are the most important aspects of your background or identity?

9. Are there any aspects of your background or identity that make a difference to your [PROBLEM]?

10. Are there any aspects of your background or identity that are causing other concerns or difficulties for you?

Now we are going to move on to the 3rd domain of this interview. We are going to focus on how you cope with your situation.

3 DOMAIN: CULTURAL FACTORS AFFECTING SELF-COPING AND PAST HELP-SEEKING

3.1 SELF-COPING

11. Sometimes people have various ways of dealing with problems like [PROBLEM]. What have you done on your own to cope with your [PROBLEM]?

SQ3:

Have you sought help for your [PROBLEM] on the internet, by reading books, by viewing television shows, or by listening to audiotapes, videos, or other sources? If so, which of these? What did you learn? Was it helpful?

SQ4:

Do you engage by yourself in practices related to a spiritual, religious or moral tradition to help you cope with your [PROBLEM]? For example, prayer, meditation, or other practices that you carry out by yourself?

3.2 PAST HELP-SEEKING

12. Often, people look for help from many different sources, including different kinds of doctors, helpers, or healers. In the past, what kinds of treatment, help, advice, or healing have you sought for your [PROBLEM]?

> **SQ2:**
>
> Have you had difficulties with clinicians in the past? What did you find difficult or unhelpful?

PROBE IF DOES NOT DESCRIBE USEFULNESS OF HELP RECEIVED:

What types of help or treatment were most useful? Not useful?

3.3 BARRIERS

13. Has anything prevented you from getting the help you need?

PROBE AS NEEDED:

For example, money, work or family commitments, stigma or discrimination, or lack of services that understand your language or background.

Now, we are in the final set of 3 questions. Now let's talk some more about the help you need.

4 DOMAIN: CULTURAL FACTORS AFFECTING CURRENT HELP-SEEKING

4.1PREFERENCES

14. What kinds of help do you think would be most useful to you at this time for your [PROBLEM]?

15. Are there other kinds of help that your family, friends, or other people have suggested would be helpful for you now?

> **AQ:**
>
> Hypothetically, though, if your family, friends, or others became aware of your problem and were interested in helping you, what would they recommend as helpful?
>
> **OR**
>
> **SQ8:**
>
> (IF HAS NOT TOLD FAMILY OR FRIENDS ABOUT PROBLEM): Can you tell me more about why you have chosen not to tell family or friends about the [PROBLEM]? How do you think they would respond if they knew about your [PROBLEM]?
>
> **SQ14:**

Would you like your family, friends, or others to be part of your treatment? If so, who would you like to be involved and how?

4.2 CLINICIAN-PATIENT RELATIONSHIP

Sometimes doctors and patients misunderstand each other because they come from different backgrounds or have different expectations.

16. SQ3:

Now let's talk about the help that you would like to get here. Some people prefer clinicians of a similar background (for example, age, race, religion, or some other characteristic) because they think it may be easier to understand each other. Do you have any preference or idea about what kind of clinician might understand you best?

SQ5:

What patients expect from their clinicians is important. As we move forward in your case, How can we best work together?

CONCLUSIONS

As we come to the end of this interview, thank you for sharing your experiences and thoughts with us. Do you have any questions or concerns about this interview?

OR

That concludes this interview. Thank you for your cooperation and for sharing your perspective and experiences. Do you have any questions or concerns about this interview?

[*the participant's answer*]

If you do not have another question, I will assign you a set of questionnaires before the intervention and ask you to complete the questionnaires. After you complete the questionnaires, you can access one of two interventions (ReDirection or Mi Bridge). If you are assigned to the ReDirection self-help program, you will get access to this program, and I will assign you a first module. If you are assigned to the Mi Bridge chat intervention, I will send you a suggestion of dates for the first chat meeting after you have completed the questionnaires. In any case, I will ask you to log in to your platform account and check your messages after completing the questionnaires.

List of messages and journal notes

Example 2 – *Title and brief description of CFI interviews (for booking purposes)*

Title: The Cultural Formulation Interview

Short description: The Cultural Formulation interview is a semi-structured interview with a BRIDGE project researcher conducted via chat, with an expected duration of 60 minutes. This interview aims to learn more about how clients perceive their problems, the causes of the problem, their experiences of seeking professional help, and their preferences in treatment. Participation in this interview is voluntary.

Example 3 – *An illustrative example of a Journal note - The client did not show up for the CFI interview*

Journal notes

2023-09-01 – Dominik Jozefík

The client didn't show up for the CFI interview – a message sent.

Example 4 – *An illustrative example of a message - the client did not show up for the CFI interview*

Subject: Cultural Formulation Interview: Action Required

Message:

Hi!

You didn't show up for today's Cultural formulation interview. If you want to reschedule it or have decided not to participate in the CFI interview, please message us via the platform.

I am sending you a link to log in to your account: https://www.iterapi.se/sites/bridge/

Best regards,

Bridge Project Practitioner

Example 9 – *An illustrative example of a Journal note – CFI not completed*

Journal notes

2023-09-01 – Dominik Jozefík

The CFI interview was not completed. 7 questions have been asked. Schedule a follow-up interview with the client.

Example 13 – *An illustrative example of a Journal note – CFI Interview Summary*

Journal notes

2023-09-01 – Dominik Jozefík

The client completed a CFI interview. The interview took place without any problems. The client was asked 16 core questions of the CFI. The further procedure was explained to the client, and questionnaires were assigned.
